# Multi-epitope vaccine against SARS-CoV-2 targeting the spike RBD: an immunoinformatics approach

**DOI:** 10.2144/fsoa-2023-0081

**Published:** 2024-05-20

**Authors:** Yasamin Pahlavan, Omid Yeganeh, Vahid Asghariazar, Chiman Karami

**Affiliations:** 1 Biosensor Sciences and Technologies Research Center, Ardabil University of Medical Sciences, Ardabil, 56189-85991, Iran; 2 Department of Microbiology, Faculty of Biological Sciences, North Tehran Branch, Islamic Azad University, Tehran, 16511-53311, Iran; 3 Cancer Immunology and Immunotherapy Research Center, Ardabil University of Medical Sciences, Ardabil, 56189-85991, Iran; 4 Department of Microbiology, Parasitology and Immunology, Ardabil University of Medical Sciences, Ardabil, 56189-85991, Iran

**Keywords:** B-cell, COVID-19, epitope vaccine, peptide, SARS-CoV-2, T-cell

## Abstract

**Aim:** We designed a SARS-CoV-2 epitope vaccine based on the receptor-binding domain (RBD) in virus spike protein. **Methods:** RT-PCR performed on nasopharyngeal swab COVID-19 patients. After registering RBD region in the GenBank, physicochemical parameters, secondary structure, homology modeling, 3D structure of RBD region and antigenicity were determined using ProtParam ExPASy, PSIPRED, MolProbity, IEDB and Vaxijen online tools, respectively. **Results:** B and T cell epitopes were predicted in terms of non-allergenicity and antigenicity. MolProbity analysis provided a qualitative model for RBD. The homology model showed that most of the residues are in optimal district of energy. **Conclusion:** High immunogenicity score of epitopes indicates promising candidates for the development of multi-epitope vaccines. It may help to develop an effective vaccine.

## Plain language summary

In order to identify the sequence of RBD region of S protein in SARS-CoV-2, RT-PCR test was performed on nasopharyngeal swab samples of four COVID-19 patients referred to Imam Khomeini Hospital in Ardabil. After registering the sequence of the RBD region in the GenBank database, the physicochemical parameters, secondary structure, homology modeling, 3D structure of the RBD region and antigenicity were determined using ProtParam ExPASy, PSIPRED, MolProbity, IEDB and Vaxijen online tools, respectively.

## Abstract (plain language summary)

The prediction of B cell and T cell epitopes was appropriate in terms of non-allergenic and antigenicity.

The homology model showed that most of the residues are in optimal district of energy.

MolProbity analysis provided a good quality model for the receptor-binding domain.

## Graphical abstract

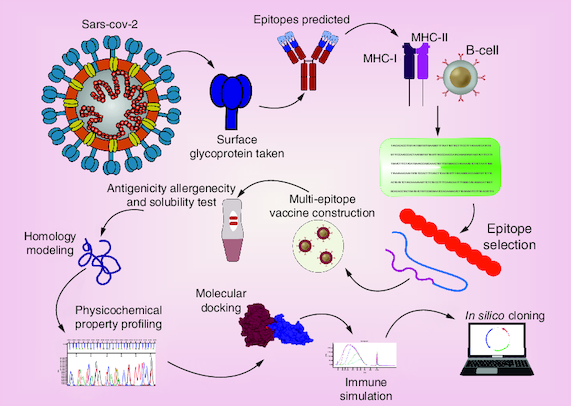

COVID-19 is the name for the pandemic condition that was at first declared as SARS-CoV-2. The COVID-19 pandemic has brought extensive concerns regarding its fast out-spread. The COVID-19 pandemic is highly pervasive and has stretched healthcare systems to their limits and has placed a huge mental, economic, and psychological burden on the entire world population. According to WHO epidemiological studies, 767,726,861 cases of COVID-19 and 6,948,764 mortality rate were reported as of 5 July 2023. Recently, 13,461,751,619 doses of various vaccines were distributed and injected in the world [1].

Pathologically, first of all, the virus appends to the receptor ACE2 on the host cell via the S1 domain of S protein. Then TMPRSS2, from serine protease family, activated the S2 domain and helped to entry the single-stranded RNA genome of virus to the lipid membrane of host cells in lung. Once the viral genome enters, a second strand is made that triggers signaling pathways to initiate innate immune responses. This stimulation activates MDA5, which is an intracellular sensor for detecting viruses. Activating the signaling pathways, JAK–STAT1/2 signaling cascade beside stimulating the expression of interferon-stimulated genes, lead to the production of interferons (IFNs) followed by systemic antiviral functions [2].

Ciliated epithelial cells are the first line of cells infected in the nasal cavity. Scientists suggest that mild and severe of COVID-19 disease caused by viruses appears in the immune system and at the level of tissue injury. Aberrant cytokine-related responses lead to functional disruption in balance from molecular mechanism to treatment and adaptive immunity to innate immunity [3].

The molecular-dynamics of multi-epitopic vaccine are considered to create a stable and protected interaction between the vaccine and the innate immune system. Introducing the virus for host innate immune system applies through pattern recognition receptors (PRRs) specially by TLR-4, *IRF3*, NF-κB and serine/threonine protein kinase of the IKK family of protein kinases that is confirmed strongly in recent studies [4]. These biomolecules are key initiators of molecular signaling pathways and activation of cytokines and regulation of gene expression that play a role in the pathogenesis of COVID-19.

Disproportionate activation of recruited immune cells to the respiratory tract as the conducting airways for the inhaled virus SARS-CoV-2 disrupts the activity of B-cells, T-cells, and natural killer (NK) cells. In addition, the pro-inflammatory state, coagulation cascade is followed by ‘thrombo-inflammation’. These perturbations result in subsequent clinical manifestations besides parenchymal damage stems including in multiple organ systems [5,6]. The emergence of the more virulent SARS-CoV-2 strains knowing the SARS-CoV-2 genetic sequence, Immunotherapy, adjunctive therapy, chloroquine or hydroxychloroquine, remdesivir, corticosteroids NSAIDs (non-steroidal anti-inflammatory drugs), monoclonal antibodies including bamlanivimab and etesevimabare used as available treatment for COVID-19 according to Food and Drug Administration issues. The latest preventative interventions vaccine against SARS-CoV-2 have been adenoviral vector-based vaccines, recombinant platforms, genetic engineering and other vaccine development technologies.

The creation of new species and its spread in the population depends on the nature of the virus and the escape from the immune system, the host's genetic background and the clinical outcome before the infection and vaccine administration. The severity of SARS-CoV-2 variant and its subvariants induced infection in different populations, has not been determined clearly. According to recent studies, there is a difference in clinical severity caused by Omicron (B.1.1.529) infection. The severity of the disease caused by Omicron BA.1 lineage is even less reported than the delta type has continued with BA.4/BA.5 [7]. This scenario represents independent and clinically significant associations BA.2 and BA.4/BA.5 lineage with risk of severe consequences. Disease severity caused by BA.4/BA.5 lineage infections compared with other Omicron lineages are less [8]. Disease burden caused by features such as ability to transmit and infect previously immune individuals, depends on vaccination or infection. Next-generation replacement of BA.4/BA.5 including XBB/XBB.1.5 as novel SARS-CoV-2 virus generations, with risk of severe illness, continue to emerge which requires preventive measures in future sciences as well as novel vaccines.

Rapid and efficient advances in the field of therapeutic strategies for COVID-19 range from small molecule-based drugs including nirmatrelvir–ritonavir, remdesivir and molnupiravir to immunomodulatory drugs such as glucocorticoids (dexamethasone), cytokine antagonists (tocilizumab), Janus kinase inhibitors (baricitinib) and monoclonal antibodies [9].

However, the lack of effective treatment for global eradication of SARS-CoV-2 has increased the global health problem. The highly transmissible nature of SARS-CoV-2 has left the entire world population with no choice but to wait for the development of a safe vaccine to break the chain of transmissible infection and contain the spread of this pandemic. Relying on conventional approaches to produce a covering vaccine is not enough. It is necessary to identify and develop the best structure for vaccine design according to protein structure and genome sequence. Today, the immunoinformatics approach has attracted attention as an ideal. One of the important structural proteins of SARS-CoV-2 is a surface protein (S protein) that acts as a spike protein, with a length of 1273 amino acids (AA) and its location is 21563–25384 AA. The S protein could be a suitable candidate for vaccine development against SARS-CoV-2 infection [10,11]. This protein contains a RBD region which enables it to bind to ACE2 and fuse into the membrane of epithelial cells. A number of newly developed vaccines have been licensed for emergency use in many countries around the world. These include mRNA vaccines, DNA vaccines, inactivated virus vaccines, and protein subunit vaccines [12,13]. Most of these vaccines usually rely on S-protein epitopes and have shown very promising results in various trial phases, but are closely monitored for any issues regarding their safety and efficacy [14]. Studies have reported that antibody responses generated against the S protein, the most prominent protein of SARS-CoV-2, can induce SARS-coronavirus neutralization and protective immunity [15].

In addition, studies have quoted that SARS-CoV-2 stimulates multiple B-cell responses which can cause virus clearance, and that CD4 T-cells help B-cells produce antibodies that lead to long-term protection [16].

The mechanisms of molecular medicine and system biology that explain a dangerous condition threatening the patient's life with systematic and coherent data in which the entire molecular network involved in the pathogenesis of COVID-19 can be justified are still unclear. The aim of this study is to design antigenic peptides derived from viral S protein for the development of SARS-CoV-2 vaccine using immunoinformatics techniques. These epitopes have the potential to raise effective responses against SARS-CoV-2. This project performed on the virus that was circulated in a local population and this is the main novelty of our study and different from other studies.

## Methods

Data sampling was performed based on the Ardabil University of Medical Sciences, Ethics Committee's published guidelines. Four swab samples were received from the Corona department and kept in a viral transmission medium for a short period of time. Swap samples kept at -80 °C until further processing and analysis. Reverse transcription polymerase chain reaction (RT-PCR) was carried out to detect the RBD region using forward and reverse primers. BigDye Terminator v3.1 Cycle Sequencing Kit from Applied Biosystems was used to sequence positive results from PCR amplification in two directions. Then, each obtained sequence was analyzed through alignment and assembly. The multiple sequence alignment of the complete amino acid sequence of each isolated SARS-CoV-2 RBD region was performed by MEGA, BioEdit and SnapGene software and compared with the sequence of different SARS-CoV-2 variants available in GenBank. Four nasopharyngeal swab samples were considered one of the samples due to weak quality was deleted. The accession number of five sequences was as MZ312430, M Z312431, MZ312432, MZ312433 and MZ312434 in online tools (https://www.gisaid.org/) and determined as a variant of interest.

### Prediction of T-cell & B-cell epitopes

IEDB tools were used to predict the conserved sequences (10-mer sequence) from human leukocyte antigen (HLA) class I and class II of T-cell epitopes by using an artificial neural network (ANN) model. Indeed, the ANN model version 2.2 was chosen as the prediction method; it depends on the median inhibitory concentration (IC_50_). For binding analyses, all alleles were carefully selected and their length was set to 10 before prediction. Analysis of epitopes binding to major histocompatibility complex (MHC) class I and II molecules was assessed by the IEDB MHC prediction server at http://tools.iedb.org (Tables 1 & 2).

**Table 1.** The T-cell epitopes prediction.

Epitopes	Antigenisity	Allergenicity and toxicity	Accessibility prediction	Beta-turn prediction	Flexibility	Hydrophobicity
SNNRIYQTSNFRVQP	0.6622	Non	1.0	1.063	1.015	2.327
PSNCLWTLKSLLIW	0.4775	Non	1.0	0.940	0.969	-2.071
KKFLLFHKFGQRHCHYCCPSTDTDSQ	0.5375	Non	1.0	1.058	0.987	1.498
QGTNTSKQVAAPLSGVTAQKSFAL	0.7786	Non	1.0	0.985	1.015	2.621

**Table 2.** The B-cell epitopes prediction.

Epitopes	Antigenicity	Allergenicity and toxicity	Accessibility	Flexibility	Hydrophobicity
APLTPSSSENKSVPLKSFHLEKK	0.7773	Non	2.144	1.137	6.643
APLTPSSSENKSV	0.6955	Non	1.594	1.137	6.643
RGDEVRQIAPGQTGKFADYNYKLPD	1.0425	Non	2.171	1.112	6.643
VRQIAPGQTGKFAD	1.2211	Non	1.865	1.112	4.643

### VaxiJen Server for prediction of antigens & subunit vaccines

Antigenicity prediction of the RBD region was analyzed by VaxiJen with a threshold of 0.4 (http://www.ddg-pharmfac.net/vaxijen/VaxiJen/VaxiJen.html). It was used to independently predict the alignment of antigens. This made it possible to classify antigens based only on the physicochemical properties of proteins without resorting to sequence alignment. This tool predicted the antigenic probability of one or more proteins based on auto cross-covariance transformation of the protein sequence.

### Prediction of allergenicity & evaluation of solubility

Allergen FP 1.0 server and AllerTOP 2.0 server were used to predict epitopes in terms of allergenicity. The solubility of the final epitopes was determined by SolPro57 and protein-sol server.

### Toxicity-prediction

Toxicity-prediction B cells and T cells used by prediction epitope toxicity online tools “https://github.com/topics/toxicity-prediction”.

### Population coverage analysis

Population coverage for each epitope was determined by the IEDB population coverage calculation tool. The IEDB online tool was used to measure four peptides in the Iranian population at an identity threshold of 100%.

### Epitope conservancy analysis & epitope clustering results

IEDB online tool was used to predict conservancy analysis and epitope clustering. This tool computes the degree of conservancy of an epitope within a given protein sequence set at a given identity level. Conservancy is defined as the fraction of protein sequences that contain the epitope, and identity is the degree of correspondence (similarity) between two sequences. Epitope Cluster Analysis used by IEDB online tool. This tool can group epitopes into clusters based on sequence identity. A cluster is defined as a group of sequences whose sequence similarity is greater than the specified minimum sequence identity threshold.

### Physicochemical properties of amino acid sequences

Physical and chemical parameters of amino acid sequences related to RBD were predicted using ProtParam ExPASy online tool.

### The secondary structure prediction

PSI-blast-based secondary structure prediction (PSIPRED) was used to investigate RBD structure. Indeed, the PSIPRED customs artificial neural network machine learning methods in its algorithm. The secondary structure of the amino acid sequences was predicted by an online PSIPRED v3.3 tool powered by the UCL department of Computer Science: Bioinformatics Group.

### Homology modeling, 3D structure prediction

Homology modeling and 3D structure of RBD were obtained using MolProbity analysis version 4.4.

## Results

### Prediction of T-cell & B-cell epitope

The conserved immunodominant peptides binding to the MHC I and II molecules at scores equal to or less than 100 median inhibitory concentrations (IC_50_), were selected for further analyses while epitopes greater than 100 IC_50_ were eliminated (Tables 1 & 2).

Prediction of T-cell epitopes of RBD region by our study indicated that epitopes with different lengths (SNNRIYQTSNFRVQP, PSNCLWTLKSLLIW, KKFLLFHKFGQRHCHYCCPSTDTDSQ, QGTNTSKQVAAPLSGVTAQKSFAL) were considered as non-allergenicity and good antigenicity. Prediction of B-cell epitopes of RBD region (APLTPSSSENKSVPLKSFHLEKK, APLTPSSSENKSV, RGDEVRQIAPGQTGKFADYNYKLPD, VRQIAPGQTGKFAD) showed non-allergenicity, good antigenicity and good solubility. The MHCI binding predictions were made using the IEDB analysis resource NetMHCpan indicated all epitopes restricted to HLA-A*02:01 alleles.

### Population coverage analysis & epitope results

Population coverage for each epitopes was carefully determined by the IEDB population coverage calculation tool. It was shown that epitope can cover 86.94% of the human population (Table 3).

**Table 3.** Population coverage analysis.

Population/area	Class combined		
	Coverage	Average hit	pc90
In this study	86.94%	6.25	3.06
Average	86.94	6.25	3.06
Standard deviation	0.0	0.0	0.0

### Epitope conservancy analysis & epitope clustering results

The T-cell epitope conservancy analysis showed that the percentage of protein sequence identity was 100% (Table 4). The results of epitope clustering are shown in (Table 5).

**Table 4.** Epitope conservancy analysis results.

Epitopes	Length	Protein sequence matches at identity (%)	Minimum identity (%)	Maximum identity
SNNRIYQTSNFRVQP	15	100.00 (1/1)	100.00	100.00
PSNCLWTLKSLLIW	14	100.00 (1/1)	100.00	100.00
KKFLLFHKFGQRHCHYCCPSTDTDSQ	26	100.00 (1/1)	100.00	100.00
QGTNTSKQVAAPLSGVTAQKSFAL	24	100.00 (1/1)	100.00	100.00

**Table 5.** Epitope clustering results.

Cluster number	Peptide number	Alignment
1.1	Singleton	SNNRIYQTSNFRVQP
2.1	Singleton	PSNCLWTLKSLLIW
3.1	Singleton	KKFLLFHKFGQRHCHYCCPSTDTDSQ
4.1	Singleton	QGTNTSKQVAAPLSGVTAQKSFAL

### Physicochemical properties of amino acid sequences

The physicochemical properties of 344 amino acids in the RBD region (Figure 2) of SARS-CoV-2 isolations were analyzed and predicted by the online tool of Expasy ProtParam bioinformatics (Table 6). The atomic composition of this region indicated a total number of atoms: 5620 with formula: C1773H2861N493O476S17. The estimated half-life is: 2.8 h (mammalian reticulocytes, *in vitro*) and 3 min (yeast, *in vivo*) and 2 min (*Escherichia coli*, *in vivo*). Theoretical pI was 10.07 based on Expasy analysis, total number of negatively charged residues (Asp, Glu) was 21, and total number of positively charged residues (Arg, Lys) was 55. The instability index was computed 43.88, which classifies the protein as unstable. Extinction coefficients are in units of M-1 cm-1, measured at 280 nm in water. Extinction coefficient 55305 Abs 0.1% (= 1 g/l) was 1.409, assuming all Cys residues form cystines. Extinction coefficient 54430, Abs 0.1% (= 1 g/l) was 1.387.

**Figure 1.**
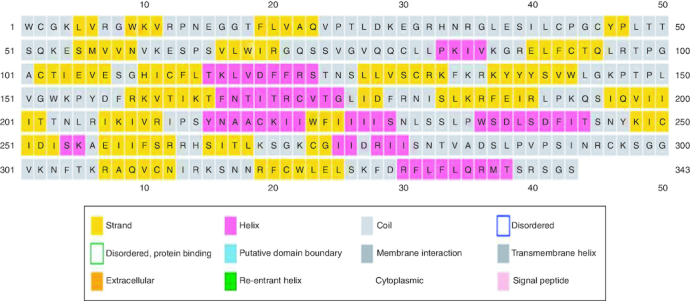
Secondary structure prediction of the RBD region of SARS-CoV-2.

**Figure 2.**
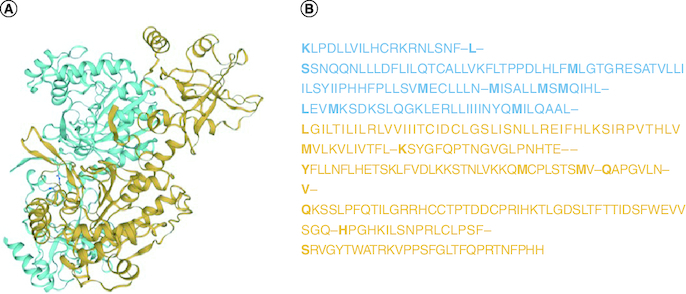
Structure of the SARS-CoV-2 spike receptor-binding domain. **(A)** 3D structure of receptor-binding domain region of SARS-CoV-2. **(B)** Nucleotide sequence of receptor-binding domain region in S gene.

**Table 6.** Physicochemical properties of amino acid sequences of the RBD region.

Target	Amino acids (n)	Theoretical pI	Negatively charged residues (n)	Positively charged residues (n)	Extinction coefficient	Estimated half-life	Instability index	Aliphatic index
RBD region	344	10.07	21	55	55305	2.8 hours	43.88	98.23

### Toxicity-prediction

Toxicity prediction of B-cells and T-cells epitopes is performed by online tools such as ‘https://github.com/topics/toxicity-prediction’. It was shown that all of them have non-toxin properties.

### The secondary structure prediction

Secondary structure prediction indicated a low-frequency of α-helix and a high rate of β-sheet in the protein structure. The secondary structure of RBD region is presented in Figure 1.

### Homology modeling & 3D structure prediction

MolProbity provided globally and locally an assessment of model quality for RBD region.

The homology model revealed that most residues were in optimal areas of energy. The MolProbity score of RBD was 2.17 and its Clash score was 7.04. The Ramachandran favored was 84.85% and Ramachandran outliers was 1.43%. The C-Beta deviations were, bad bonds 5.602 and bad angel 5.806 and twisted prolines 1.2, QM -6.71. The prediction of the third structure of RBD is depicted in Figure 2.

## Discussion

When SARS-CoV-2 spread around the world in December 2019, nobody thought that these pandemic conditions might continue for more than one year. The world has been exposed to more mutations which have led to changes in virus molecular structure with possibly more transmission ability of the disease in the human population [17]. Overall, vaccination is still the most economical and effective way to eradicate virus infection [18]. The selection and design of protective immunogens against microorganisms is a significant manner in vaccine development, especially for newly emerging pathogens [19]. Here we proposed a SARS-CoV-2 epitope vaccine concept based on the identification of conserved peptide regions of the viral genome and newly acquired alterations. We further presented genomic regions that predicted T-cell and B-cell epitopes. These contain peptides in RBD regions of the spike protein that have been reported to enhance infection by binding to the ACE2 protein and are thought to intensify membrane fusion. Validation and administration of this epitope-based vaccine may address the specific vulnerabilities of COVID-19 and should enhance a strong adaptive immune response in the vast majority of the population [20]. Regardless, the nature of the SARS-CoV-2 virus is variable, and sequence analysis of the RBD may reveal distinct differences among human populations that are differed by previous infections and genetic variation, leading to immune responses and disparities in risk of COVID-19. In the present study, the homology model revealed that most residues were in the optimal district of energy. Secondary structure prediction indicated low-frequency α-helix and a high rate of β-sheet in protein structure. It is worth mentioning that the abundance of β-sheet in the protein structure can reduce the flexibility of the protein and increase the resistance to degradation [21]. Also, T-cell epitope conservancy analysis showed that the percentage of protein sequence identity was 100%. In this regard, conservancy is defined as the fraction of protein sequences that contain the epitope, and identity is the degree of correspondence (similarity) between two sequences. Population coverage analysis with identity threshold 100% showed that epitopes with a minimum length of 10 nucleotides can cover 86.94% of the human population. The procedures described here may provide an appropriate schema for the assessment of immunogenic regions of viral capsid protein for use in vaccination. It indicates that an immune response to the epitopes mentioned here may provide broader protective immunity against mutant variants of SARS-CoV-2 and other coronaviruses as well as other microorganisms [22]. In this study, we characterized epitopes that contain newly acquired features of SARS-CoV-2 that confer evolutionary advantages in viral mutation and infectivity [23]. Traditional vaccines such as attenuated viral materials, although historically successful in protecting against viral diseases, require long-term cell culture operations to obtain the attenuated strains. However, epitope-based peptide vaccines require *in vitro* culture and show the ability to induce stronger memory responses with fewer immunization side effects [24,25]. It is assumed that the immunogenicity of epitope vaccine presented here can be used by the scientific community for preventive measures against future outbreaks. In accordance with our study, researchers designed multiepitope-based vaccines derived from lymphocyte epitopes against SARS-CoV-2 [26]. Other studies predicted that protective epitopes can bind to MHC classes, indicating excellent antigenicity and good surface accessibility. Researchers proposed a SARS-CoV-2 vaccine design based on epitopes presented on MHC class I and II in human cells. Researchers also screened T-cell and B-cell epitopes of S protein based on the antigenicity, toxicity, allergenicity, and cross-reactivity with human proteomes [27]. A peptide-based vaccine using spike glycoprotein has been reported as non-toxic and non-allergenic to stimulate better immunological responses with acceptable safety [28].

These findings were extended and potential B-cell and T-cell epitopes were predicted for SARS-CoV-2 by parallel bioinformatics [29]. Given the recent release of the Omicron strain of SARS-CoV-2 and the emergence of other possible variants worldwide, introducing effective vaccines and/or approved treatment is definitely a high priority. Our analysis was performed on B-cell and T-cell epitopes deduced from SARS-CoV-2 (S) protein with high antigenicity and no allergenic or toxic effects. Due to the limitations and high cost of sequencing, only five samples were sequenced. Out of these five samples, one of these samples was excluded from the study due to the low quality of the sequence, and only four samples were analyzed. These findings suggest a set of peptides that can be used as suitable targets for the development of epitope-based vaccines against SARS-CoV-2 virus. Other aspects of SARS-CoV-2 virus or different immunoinformatics approaches, which have not yet been widely studied, are data that can explain signaling pathways in addition to molecular and medical contributions. In our project, in order to obtain the originality of study to cover a small part of these approaches, we used a bioinformatics approach on SARS-CoV-2 that was spread in the local population.

## Conclusion

The acquisition of immunological data has given rise to the field of immunoinformatics, which provides insights into the mechanisms of immune function. Epitope information presented by this study may help in developing an effective vaccine against SARS-CoV-2. This will help identify SARS-CoV-2 epitopes for a pan-coronavirus vaccine to prepare for future pandemics. Recent advances in vaccine platforms depend on population genomics and immunoinformatics resources and the prospect of advanced personalized medicine in using technology alongside antibody/nanobody origins to control current virus and enhance the efficacy of immune responses.
